# The motherhood role expectations for young female patients with breast cancer: a qualitative study

**DOI:** 10.3389/fgwh.2026.1750318

**Published:** 2026-06-12

**Authors:** Yingrui Han, Wenjuan Gao, Qian Zhang, Dan Wang, Xiaoxu Li, Na Deng, Li Ma, Jing Han

**Affiliations:** 1 School of Nursing, Xuzhou Medical University, Xuzhou, Jiangsu, China; 2 Department of nursing, Affiliated Hospital of Xuzhou Medical University, Xuzhou, Jiangsu, China; 3 Department of nursing, Xuzhou Central Hospital, Xuzhou, Jiangsu, China

**Keywords:** breast cancer, motherhood, qualitative study, role expectation, young woman

## Abstract

**Background:**

Breast cancer critically disrupts young mothers’ capacity to fulfill motherhood role functions, while reshaping family expectations. There is a lack of research on the expectations of motherhood after diagnosed with breast cancer among young women. This study aims to explore young Chinese breast cancer patients’ motherhood role expectations from multiple perspectives, including patients’ and family members’ voices.

**Methods:**

This study recruited participants via purposive sampling, including 20 young women with breast cancer, 6 spouses, and 4 children. All participants took part in face-to-face individual semi-structured interviews. The Colaizzi descriptive analysis method was used to analyze the interview data.

**Results:**

Two core dimensions of motherhood role expectations were identified by this study among young women with breast cancer and their families: self-oriented expectations and parental responsibility expectations. Self-oriented expectations cover two key themes: safeguarding life and maintaining the dignity of motherhood. Parental responsibility expectations include two themes: taking care of the child like a normal mother and effectively addressing breast cancer-related challenges.

**Conclusion:**

For young breast cancer patients and their families, expectations regarding the patient's role as a mother revolve around two key aspects: the patient's own health and the discharge of parental responsibilities. Although the patient's health condition forms the foundation of realizing these motherhood role expectations, the ability to cope with the multifaceted challenges related to breast cancer constitutes a unique requirement for them to fulfill this role after diagnosis.

## Background

Young adults with breast cancer are defined as those diagnosed before the age of 40 (1). Globally, breast cancer is the single most common malignancy among this population, accounting for 15% of all cancers in both the United States and China (2, 3). Young adults with breast cancer are more frequently diagnosed at an advanced stage of the disease; they also face a higher risk of breast cancer recurrence and death than older patients (2, 4). Current evidence confirms that active treatment, including surgery, chemotherapy, and radiotherapy, effectively reduce recurrence rates and mortality in young adult patients with breast cancer (5, 6). Compared with older patients, young adults with breast cancer are at higher risk of depression, poor body image, occupational difficulties, concerns about sexuality and fertility, and financial toxicity (7). Meanwhile, these patients are of childbearing age, and global statistics indicate that one-third of these patients are parents of younger children at the time of their diagnosis (2, 8).

Young breast cancer patients experience heightened stress as they juggle cancer treatment and motherhood responsibilities. Studies show that after diagnosis, these patients report higher levels of stress and anxiety compared to postmenopausal patients and those without children, respectively (9, 10). Previous studies have indicated that patients with breast cancer frequently encounter significant challenges in their motherhood roles, such as childcare (11), cancer-related communication (12), managing negative emotions (13). Treatment demands and physical impairment often drastically reduce the time available for childcare (11). Li et al. found that 40% of patients undergoing radiation therapy encountered childcare conflicts, while 14% had to reschedule or miss at least one treatment appointment (14). Caparso et al. found that parents with cancer always struggle initiating conversations with their children about the cancer, and desire assistance about addressing mental health concerns and emotions (12).

Suffering during breast cancer is not limited to the woman, but extends to the family system. Existing research demonstrates that spouses experience significant caregiver burden, anxiety, depressive, and fatigue (15, 16). Milbury et al. revealed spouses exhibited higher parenting stress and anxiety compared to patients. Despite patients’ conscious efforts to maintain normative parenting, their children always reported high distress. A systematic reviews estimated that 22%–33% of this population scores within or above the clinical range on validated psychosocial adjustment scales (17). Almulla et al. found that children's predominant concern was the fear of their parent's cancer-related death, even when the disease was diagnosed at an early stage (18).

Role Theory posits that social roles comprise patterned behaviors, assumed identities by social participants, and shared behavioral expectations that are understood by all and adhered to by the performer (19). In Asian cultures, motherhood is traditionally characterized by three core functions: childcare, educational guidance, and emotional support (20). Breast cancer critically disrupts young mothers’ capacity to fulfill these expectations (21), while dynamically reshaping family expectations. Studies indicate spouses often assume disproportionate caregiving duties (22), and children develop altered perceptions of maternal abilities (18). This role renegotiation would exacerbate patients’ psychological distress through guilt. However, existing studies on breast cancer patients have predominantly focused on their negative motherhood role experiences, leaving a critical gap in exploring their role expectations from a positive perspective. This exploration is practically essential for patients and families, as it can help them balance “patient” and “mother” identities and alleviate role conflict.

The aim of this study was to explore young Chinese breast cancer patients’ motherhood role expectations by centering patients’ and family members’ voices—a multi-perspective approach enhancing finding comprehensiveness. The results are expected to: help patients cope with maternal dilemmas; deepen health professionals’ and social workers’ understanding of this group's maternal needs; and guide supportive interventions for patients and families.

## Methods

### Design

The descriptive phenomenology is used to explore the motherhood role expectations of patients with breast cancer, which can help researchers to reveal the essence and meaning of lived experience and to describe the basic structure of the phenomenon of life experience (23).

### Participants and recruitment

Participants in this study included young patients with breast cancer and their family members. For patients, inclusion criteria were: (a) < 40 years old, (b) being diagnosed with breast cancer first time, (c) having no language or cognitive impairments, (d) having one or more dependent children, and (e) voluntary participation with signed informed consent form; and exclusion criteria were: (a) having a history of other cancers, (b) being diagnosed with a psychiatric disorder previously. For family members, inclusion criteria were: (a) being the spouse or child of the enrolled patient, (b) knowing of the patient's breast cancer diagnosis and disease status, (c)having basic reading skills and normal verbal communication abilities, and (d) voluntary participation.

Purposive sampling was employed to recruit participants from the Breast Center of a tertiary hospital with 4,150 beds, located in a coastal city in eastern China with a permanent population of 9 million. Researchers distributed study information sheets to eligible young breast cancer patients and invited them to participate, while also extending invitations to the patients’ spouses or children.

### Data collection

Data were collected using a semi-structured interview guideline (see Table 1). This guideline was developed via an extensive literature review and consultation with an oncology psychologist. Prior to formal data collection, two pilot interviews were conducted with patients to refine the interview questions.

**Table 1:** The semi-structured interview guideline.

●	Young breast cancer patients
Q1	Please think about how your illness has affected your role as a mother.
Q2	What are your expectations as a mother after you get the disease?
Q3	What do you think you should do for your children after you get sick?
Q4	What do you think is the most important thing to be a mother after illness?

●	Family member
Q1	Please think about the changes in her motherhood after the illness.
Q2	What expectations do you have of your ill wife/mother in her role as a mother?
Q3	What do you think was the most important thing she did as a mother after her illness?
Q4	What mother role duty would you like her to fulfil afterwards? Please be specific.

The data collection process comprised three steps, as detailed below: (a) First, participants were informed of the study's purpose and significance. They then signed the informed consent form and the authorization for interview recording. For participants under 18 years of age, their legal guardians (parents) signed the informed consent form on their behalf. (b) Second, face-to-face, semi-structured individual interviews were conducted in the center's interview room. Each interview involved only the participant and the interviewers (DW & LM). During interviews, the researchers documented participants’ non-verbal behaviors (e.g., facial expressions, intonation, and emotional states) in writing. (c) Notably, data collection and analysis were conducted concurrently. Data collection was terminated when data saturation was achieved—defined as the point at which no new information emerged from interviews (24).

### Data analysis

The records were transcribed within 24 h after the interviews. Transcription was performed by one researcher (QZ), while the other researcher (WG) checked the consistency of the text and records. The three researchers (YH, WG & QZ) used Colaizzi's seven-step method to analyze the data: (a) transcribing all descriptions of the subjects; (b) extracting significant statements; (c) creating formulated meanings; (d) aggregating formulated meanings into theme clusters; (e) developing an exhaustive description; (f) identifying the fundamental structure of the phenomenon; and (g) returning to participants for validation. Constant comparisons between data and memo were performed during coding (25). The codes were cross-compared, and narratives sharing similar codes were collated and presented from the patients’ and families’ viewpoints. Any inconsistent codes and emerging issues were thoroughly discussed and amicably resolved within the research group. Through a profound and in-depth analysis of the participants’ narratives, the key themes and essential elements related to the expectations of motherhood role were extracted.

All researchers are nurses trained in qualitative research with oncology clinical experience. As the two interviewers (DW & LM) who are also mothers, we recognized how maternal perspectives could influence data collection and interpretation. To enhance reflexivity, we employed bracketing to actively distancing the researcher from the various relationships during the interview and analysis process (26). To surface potential biases, they (DW & LM) wrote a pre-study memo stating initial assumptions (e.g., that a breast cancer diagnosis would severely disrupt mother-child bonding), which were then discussed within the research team. During analysis, the researchers wrote reflective memos to suspend preconceived ideas (26). The analysis team (qualitative methodologists, nurse specialists, and interviewers) met biweekly to compare independent coding, challenge interpretations, and document how their different backgrounds shaped theme development. Disagreements were resolved by discussion, with decisions recorded in an audit trail. For child participants, we conducted a brief debriefing after each interview to ensure interpretations remained grounded in the child’s responses.

The data were analyzed in Chinese. When summarizing the results and writing the manuscript, the two researchers translated the text into English using the example quotations from the database. Two bilingual nurses with expertise in qualitative research reviewed the manuscript to ensure the translation accuracy. Where there was a lack of clarity, discussions were held between the research team and the translation experts to ensure that the intended meaning of the text was retained to the greatest possible extent.

### Ethical considerations

This study was approved by the ethical committee of the Affiliated Hospital of Xuzhou Medical University (Approval number: XYFY2022-KL452-01). All procedures performed in this study were in accordance with the ethical standards of the institutional and national research committees and with the 1964 Helsinki declaration and its later amendments or comparable ethical standards. All participants were informed that the interview data would only be used in this study and all personal information would be concealed. Formal written informed consent was obtained before the interviews commenced. All participants were informed that they could refuse to answer any questions or withdraw from the study at any time, and that would not affect their treatment and care.

For child participants, we obtained parental written consent and child verbal assent. The ethics committee approved the inclusion of minors under the same approval number. A child-friendly information sheet was used. The interviewer remained available to answer any questions and monitored for signs of distress, with a plan to refer to a counselor if needed.

### Rigor

Four main criteria are used to assess the trustworthiness of qualitative research: credibility, transferability, dependability, and confirmability. In this study, credibility was enhanced through peer debriefing and member checking. Peer debriefing involved a series of discussions between the two researchers. Disagreements about codes led to improvements through consensus. Member checking was performed by senior researchers (XL & JH). For transferability, the researchers described the participants’ demographic characteristics and interview guidelines in detail to help readers recognize the relevance of the results in other contexts. To increase dependability, information-rich data were analyzed, and four researchers (RH, WG, QZ & ND) were involved in the data analysis process. Throughout the process of data collection and analysis, the researchers suspended their possible preconceptions, personal thoughts, and feelings to objectively and fully immerse themselves in participant experiences, and compare the memo with the original data. For confirmability, the findings were returned to the participants who all confirmed the accuracy of the findings.

## Results

### Characteristics of participants

From March to August 2024, 22 participants were recruited. However, two participants could not be interviewed for medical reasons. Study participants therefore finally included 20 patients (mean age 35.6 years), 6 spouses (mean age 38.4 years), and 4 children (mean age 14.5 years). Spouses and children come from different families of the recruited patients. Details of the participants are shown in Table 2. The interview duration ranged from 32 to 69 min.

**Table 2:** Demographic characteristics of the participants.

Variables	n	%
Patients characteristics (*n* = 20)		
Marital status		
Married, living together	19	95
Divorced	1	5
Education level		
Low (≤9th grade)	12	60
High (>9th grade)	8	40
Number of children		
1	10	50
2	10	50
Age of children		
≤6	9	30
7∼12	7	23.3
13∼18	14	46.7
Disease Stage (TNM)		
Ⅰ	3	15
Ⅱ	11	55
Ⅲ	6	30
Current stage of treatment		
Active treatment		
Operation	6	30
Chemotherapy	10	50
Radiotherapy	2	10
Standardized monitoring phrase	2	10
Spouses characteristics (*n* = 6)		
Education level		
Low (≤ 9th grade)	4	66.7
High (>9th grade)	2	33.3
Children characteristics (*n* = 4)		
Junior middle school	2	50
Senior middle school	2	50

### Expectations of the motherhood role of young breast cancer patients

**Figure 1:**
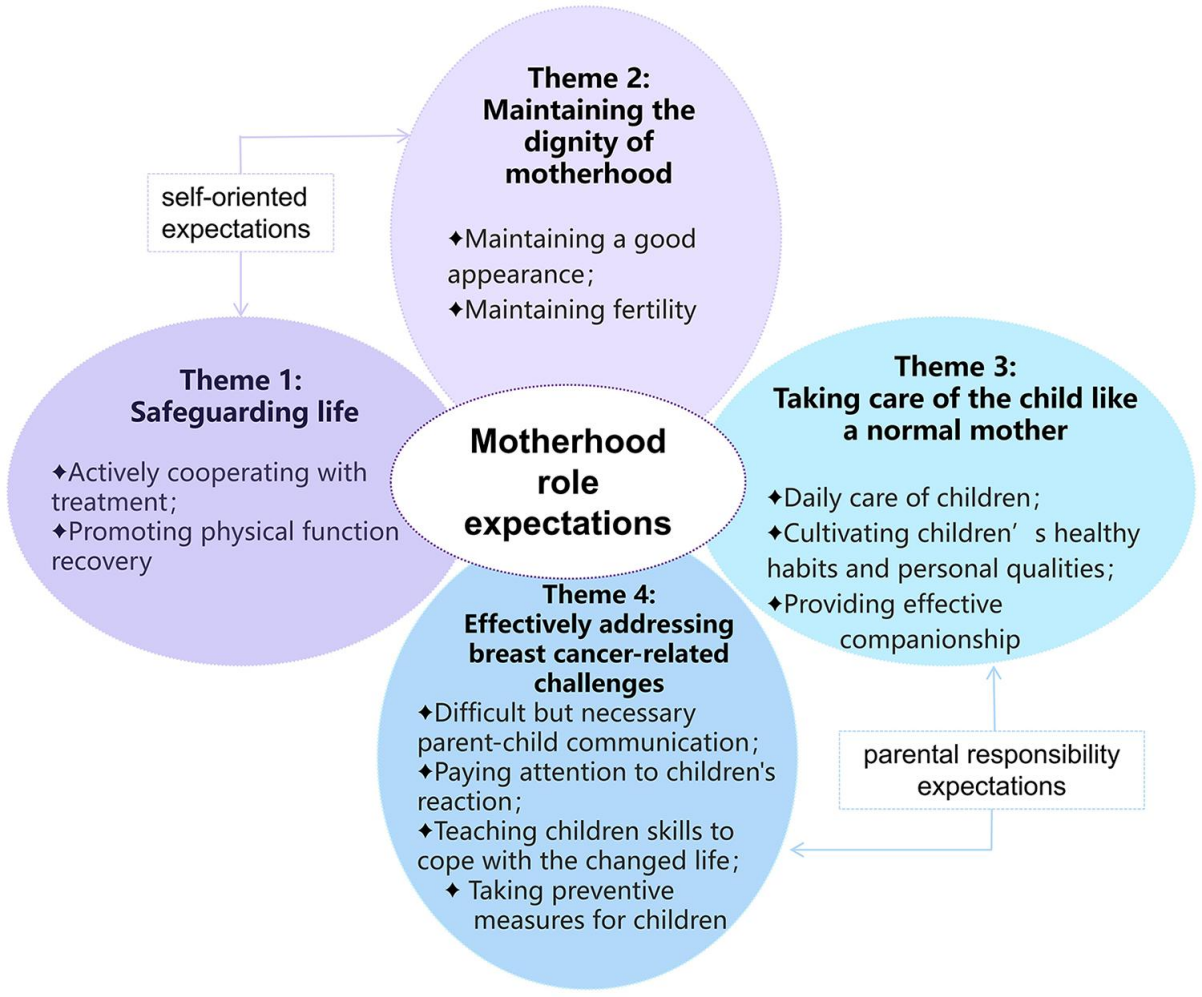
Motherhood role expectations for young breast cancer patients.

This study identified two core dimensions of motherhood role expectations for young women with breast cancer: self-oriented expectations and parental responsibility expectations. Self-oriented expectations encompass two themes: safeguarding life and maintaining the dignity of motherhood. Parental responsibility expectations include two themes: Taking care of the child like a normal mother, and effectively addressing breast cancer-related challenges (see Figure 1). Details of themes, sub-themes, and code counts are presented in Table 3. The coding process is described in the Supplementary ‘Coding Process’.

**Table 3:** The themes, sub-themes, and number of codes.

Themes	Sub-themes	Number of coding material sources
Safeguarding life	Actively cooperating with treatment	16 (patients); 5 (spouses); 4 (children)
	Promoting physical function recovery	14 (patients); 3 (spouses); 2 (children)
Maintaining the dignity of motherhood	Maintaining a good appearance	12 (patients)
	Maintaining fertility	13 (patients); 2 (spouses)
Taking care of the child like a normal mother	Daily care of children	17 (patients); 2 (children)
	Cultivating children's healthy habits and personal qualities	11 (patients)
	Providing effective companionship	15 (patients)
Effectively addressing breast cancer-related challenges	Difficult but necessary parent-child communication	16 (patients); 6 (spouses); 3 (children)
	Paying attention to children's reaction	12 (patients); 3 (spouses)
	Teaching children skills to cope with the changed life	9 (patients); 2 (spouses); 2 (children)
	Taking preventive measures for children	16 (patients); 5 (spouses); 2 (children)

### Theme 1: safeguarding life

When faced with a breast cancer diagnosis and the role of motherhood, the first expectation of patients and families is to safeguard life. This is because breast cancer threatens the patient's life and health. Safeguarding life is the basis for being able to be with and care for their children longer. So they expect to live longer by being active in their treatment and by exercising.

#### Sub theme 1.1: actively cooperating with treatment

Faced with the threat of breast cancer, patients must be forced to undergo a variety of treatment options and go to the hospital on time to prolong their life. This is the basis of their companionship and care for their children. Their families are also eager for the patients to actively cooperate with the doctors in their treatment endeavors.

“I will go to the hospital on time for chemotherapy in order to survive and be with my child longer.” (P4) “The biggest thing I expect from my mum is that she listens to the doctors and goes to the hospital on time.” (P7 son)

#### Sub theme 1.2: promoting physical function recovery

Patients want to recover as quickly as possible by eating a healthy diet, getting enough sleep, and undertaking physical exercises appropriately for their health. They see their children as their motivation to promote recovery. The patient's family also monitors their lifestyle habits in the hope of a speedy recovery.

“Eating healthier now. I have to live a few more years for the sake of my husband and children.” (P13) “I'm hoping that I can recover sooner rather than later and do more exercise moves when I'm not tired so that I can lift my arms.” (P3) “I will supervise my mother and do sports at home every day. ” (P8 daughter)

### Theme 2: maintaining the dignity of motherhood

Maintaining the dignity of motherhood is very important to the patient's self. They do not want their image in their children's minds to be affected by the various changes brought about by the treatment. So they attempt to cover up their discomfort in various ways to maintain a good appearance. In addition, breast cancer treatment interrupts the plans of having another child. They desire to maintain their fertility.

#### Sub theme 2.1: maintaining a good appearance

Patients feel sad in the face of all the discomfort caused by the treatment. They try to hide their physical and psychological discomfort from their children in order to prevent the beautiful image of motherhood from being destroyed. Patients could not bear the thought of their children seeing them without hair. So they wear hats or wigs. They also dress up when they go out with their children to maintain dignity and pride.

“When the kids are at home, I wear a hat. After all, I'm bald now. Children will be scared to look at it, I still want to have a better image of their mother.” (P5) “I now wear a wig and make-up when I go out with her (my daughter). I can't look too bad. I can't embarrass my child.” (P5)

#### Sub theme 2.2: maintaining fertility

Some of the patients had planned to have another child before they were diagnosed with breast cancer, but had to postpone or abandon these plans because of treatment. They expressed frustration and felt that the breast cancer had robbed them of their right to become mothers again. The spouses felt the same way but were afraid to talk about this topic with their ill wives. In addition, many patients expected to maintain fertility and hoped that medical professionals could provide relevant advice.

“I have been planning to have a second child. But after diagnosed cancer, it seems that I can't have a child again. I'm disappointed. But I still hope to have a child. I don't have the specific knowledge either. I hope you can tell me more.” (P12) “Because she was sick, she was afraid to have a second child. Actually I still want to have another child. But now I don't dare to say it in front of her (wife) either, for fear of increasing her mind burden.” (P12 spouse)

### Theme 3:taking care of the child like a normal mother

Despite their illness, young breast cancer patients want to fulfill their parental responsibilities by taking care of the child like a normal mother. Patients want to provide as much daily care for their children as physically possible and to cultivate children's healthy habits and personal qualities. In addition, the sudden onset of the disease made the patients realize the importance of providing effective companionship for their children. They want to spend more time with their children and enjoy parent-child time.

#### Sub theme 3.1: daily care of children

This is even though they are ill and the rhythm of lives has been disrupted. However, the patients still wanted to be able to continue providing their children with day-to-day care, to continue carrying out daily care for their children, participating in the children's school activities, and ensuring children's daily expenses, as much as possible. At the same time, the children also wanted their daily lives not to be significantly altered by their mother's illness.

“I want to be along with her at school activities (daughter). After dismissing chemotherapy, I will return to work to earn money for her study and living.” (P2) “I prefer to eat my mum's cooking. I hope she can cook for me in the future. Occasionally she could accompany me to sleep. Don't change that.” (P12 son)

#### Sub theme 3.2: cultivating children’s healthy habits and personal qualities

After the illness, patients realized the importance of good health. Patients pay particular attention to developing good habits in their children, such as healthy eating habits, balanced study and rest, appropriate physical exercise, and better study habits.

“I told him (my son) the reason why I was sick, which may be related to my lack of exercise and poor health. I will tell him to do physical exercise, eat healthily, and get a good sleep so that he won't get sick. ” (P5)

Patients see illness as a good opportunity to educate their children. They used their illness to develop a sense of responsibility in their children, to build their resilience to stress, to guide them to express themselves better, to develop their sense of gratitude, and to encourage them to face life positively. Patients want to give children healthy personal qualities.

“It just so happens that he (his son) is a little bit older and it's time to train him to be responsible. Since I've been sick he's been taking care of me with my dad, and I told him you're a man now, and now that mummy's down, you're going to help dad share the load.” (P2) “I will teach my children to be positive. You see I don't have a lot of reactions when I take this medicine (chemotherapy medicine) to the doctor because I have a good mindset.” (P8)

#### Sub theme 3.3: providing effective companionship

Although patients needed to spend much time and energy on treatment and rehabilitation, they hoped to accompany their children effectively when their bodies allowed, such as reading together and traveling. Through providing effective companionship, the parent-child relationship can be strengthened, and at the same time, the children's horizons can be broadened.

“I'm just going to wait until I'm done with this chemo. Ask the doctor if I can just take the kids out for a trip when I'm better, and not too strenuous. Because now the kids are on holiday, I want to take them to the beach.” (P18)

### Theme 4: effectively addressing breast cancer-related challenges

Young breast cancer patients and families have special expectations for the role of the mother, which can be summarized as effectively responding to the challenges brought by cancer. They feel it is necessary to inform children of the fact that mother is ill. However, the choice of how to do this is very difficult. Also, after informing the children, their reaction is something that needs to be carefully observed. Moreover, the treatment brings more life changes and mothers must teach their children some coping skills. In addition, preventing the inheritance of the disease is considered by the mother to be of the utmost importance in maintaining children's health.

#### Sub theme 4.1: difficult but necessary parent-child communication

Patients and their spouses consider it necessary to proactively inform their children about illness. However, communication decision-making was difficult. On the one hand, patients wanted to protect their young children from being scared by their cancer, and on the other hand, patients did not know how to tell their children about their disease information. Therefore, based on the children's cognitive level, the patients provided the necessary information related to breast cancer and treatment to minimize children's concerns.

“It is necessary to tell my child of my condition, my sickness, my sad emotion truthfully. However, the communication process was difficult. It is difficult to tell these problems. How to tell? I thought of many methods and took a long time to tell my little daughter about my condition so as not to hurt and scare her so much. I felt that my child had a correct understanding of the disease and my condition” (P7) “I want her (mum) to be honest and tell me everything about her illness.” (P8 daughter)

#### Sub theme 4.2: paying attention to children’s reaction

After informing the children of the disease, their reaction is also crucial. Patients and spouses make appropriate reassurances based on their children's reactions. And they are happy to notice some changes in their children, such as improved ability to take care of themselves.

“Ever since I told her (my daughter) I was sick, I've been very concerned about her reaction and worried that she might be scared. Because she’s still very young. But I've noticed that she’s starting to look after herself and needs as little help from me as possible. I felt very happy about that.”(P14)

#### Sub theme 4.3: teaching children skills to cope with the changed life

Because the patient requires frequent hospitalization, this also changes the children's daily lives. The patient was concerned that without the mother's care, the children would have a lower quality of life and experience emotional changes. To cope with this challenge, the patient started to try to communicate with her children, understand their feelings, and teach them life skills and independent learning.

“I will chat with my child more and let him not be too sad.If he looks sad, we will watch TV or walk along the river. This makes him feel better. Because his father needs to go out to work, I am in the hospital and the children are left unattended. Therefore, I have taught him to cook and write his homework by himself. ” (P15)

#### Sub theme 4.4: taking preventive measures for children

The heredity of breast cancer is the most worrying problem for patients, which makes them feel guilty about their children. Patients (especially those with daughters) felt that they needed to take preventative measures to minimize their children's risk of developing breast cancer, such as genetic testing to determine if their breast cancer is hereditary, to keeping their children up to date with regular breast exams, and for keeping their children up to date with breast cancer vaccines when they become available in the future.

“I gave myself a genetic test to see if it is hereditary, and I will take my daughter for an annual health examination in the future. If there is a vaccine to prevent breast cancer, I will let her get it.” (P11)

## Discussion

To our knowledge, this study is the first to describe expectations of the motherhood role from the women with breast cancer and families. When interviewed separately, patients and families are expressing similar expectations, which is the main finding of this study. For young women with breast cancer, these expectations were divided into two key dimensions, namely self-related expectations and parental responsibility-related expectations. In the self-related dimension, the most fundamental shared expectation is safeguarding the patient's life and maintaining the dignity of motherhood role. In the parental responsibility-related dimension, there is consistent two expectation: taking care of the child like a normal mother and effectively addressing breast cancer-related challenges.

Safeguarding life was the primary and basic motherhood role expectation of young breast cancer patients, and patients and families considered the patient role before the motherhood role. In Asian culture, mothers always put their children first and tend to neglect their own needs (27). Breast cancer makes patients reflect on the important things in life, and become care and concerned for their health. Nizamli's study also found that breast cancer patients expressed the need for treatment during the disease, thinking first and foremost about their physical and emotional problems (28). Although the patient role has the right of exemption from social responsibility (29), and the priority for women with breast cancer in the patient role is to cooperate with treatment (30), patients would feel guilty about the burden their illness imposes on their husbands and children (31). This finding guides clinical nursing for young breast cancer patients in two key ways: nurses should prioritize the “patient role” in care while addressing role guilt via family communication and role adaptation education.

Maintaining the dignity of motherhood is another motherhood role expectation. When being with their children, patients would compensate for changes in their appearance by wearing hats and applying makeup. They do this to look “normal,” reduce others’ curious or unkind stares, and in turn safeguard their children's dignity. Meanwhile, participants expressed a strong desire to preserve fertility. In Asian cultural traditions, there is a deeply rooted belief that a larger family contributes to greater well-being. Although adoption could allow them to become mothers again, couples in Asian cultures still tend to prefer having biological children. However, breast cancer treatments such as surgery, chemotherapy, and radiotherapy often impair women's fertility (32), forcing patients to postpone or even cancel their pregnancy plans, and this can cause them significant distress. Healthcare professionals should deliver comprehensive fertility preservation counseling tailored to young breast cancer patients and their partners (33).

Taking care of the child like a normal mother was the important expectation of parental responsibility for young women with breast cancer. This is consist with the Role Theory, poiting that the traditional motherhood finctions were childcare, educational guidance, and emotional support (20). Fisher's study also found that breast cancer patients face parenting issues such as difficulty caring for their children and inability to be present (34). Meanwhile, effectively addressing the challenges posed by breast cancer was the special expectation of motherhood role of young breast cancer patients and their families, such as parent-child communicating cancer concerns, paying attention to children's reaction, teaching children skills to cope with the changed life, taking preventive measures for children. Previous research has focused on communicating cancerrelated concerns to the dependent children of parents with cancer (35). Patients would pay attention to children's reactions and teach them to cope with changed life. This is an important step in facilitating children's adjustment to life after the illness and a more positive parent-child relationship (36). Meanwhile, patients believed that early prevention of cancer for their children was necessary. Smalls et al. found that although the majority of young breast cancer patients felt it was their responsibility to enroll their children in breast cancer screening, most of them did not put these intentions into practice due to a lack of knowledge (37). Therefore, healthcare professionals should provide communication skills and prevention information for parents to help them take on the action.

### Limitations

There are some limitations of this study. First, this study was conducted at a single institution over 6 months, with participants limited to those willing and able to actively express themselves. Additionally, while family members were included for multiple perspectives, their small number might limit the results’ representatives and generalization. Second, the patient and families’ expectations of the motherhood role may be changed according to their physical and psychological function. Future research can explore motherhood role expectations along with the disease trajectory. Third, most of the researchers are female, which may lead to analyzing and presenting the results of this study from a female perspective.

## Conclusion

The young breast cancer patients and their families have elaborated their expectations of motherhood role from the perspective of self and parental responsibility. For the patients and their families, safeguarding life and maintaining the dignity of motherhood are not only the most fundamental demands, but also the cornerstone of the whole family support system, and the necessary prerequisite for mothers to be able to continue to assume the responsibility of caring for their children. From the perspective of parental responsibility, their desire to grow up with their children as ordinary mothers do, while effectively coping with the physical and psychological challenges of breast cancer, reflects their firm adherence to their parental responsibility under special circumstances. The findings enriched the role theory related to the cancer patients, and would be useful for cancer families, healthcare professionals and social workers. The research findings can help cancer families more effectively navigate and harmonize the dual roles of patient and mother. Health professionals and social workers can provide targeted support strategies to assist patients and their families to better adapt to the challenges related to breast cancer, thereby improving their quality of life and family function, and promoting the harmony and stability of the entire family system.

## Data Availability

The original contributions presented in the study are included in the article/Supplementary Material, further inquiries can be directed to the corresponding author.
